# Effect of task-related continuous auditory feedback during learning of tracking motion exercises

**DOI:** 10.1186/1743-0003-9-79

**Published:** 2012-10-10

**Authors:** Giulio Rosati, Fabio Oscari, Simone Spagnol, Federico Avanzini, Stefano Masiero

**Affiliations:** 1 Rehabrobotics Lab, Dept. of Management and Engineering, University of Padua, Via Venezia 1, I-35131 Padova, Italy; 2 Sound and Music Computing Group, Dept. of Information Engineering, Univ. of Padua, Via Gradenigo 6/B, I-35131 Padova, Italy; 3 Rehabilitation Unit, Dept. of Neurology, University of Padua, Via Giustiniani 2, I-35128 Padova, Italy

## Abstract

**Background:**

This paper presents the results of a set of experiments in which we used continuous auditory feedback to augment motor training exercises. This feedback modality is mostly underexploited in current robotic rehabilitation systems, which usually implement only very basic auditory interfaces. Our hypothesis is that properly designed continuous auditory feedback could be used to represent temporal and spatial information that could in turn, improve performance and motor learning.

**Methods:**

We implemented three different experiments on healthy subjects, who were asked to track a target on a screen by moving an input device (controller) with their hand. Different visual and auditory feedback modalities were envisaged. The first experiment investigated whether continuous task-related auditory feedback can help improve performance to a greater extent than error-related audio feedback, or visual feedback alone. In the second experiment we used sensory substitution to compare different types of auditory feedback with equivalent visual feedback, in order to find out whether mapping the same information on a different sensory channel (the visual channel) yielded comparable effects with those gained in the first experiment. The final experiment applied a continuously changing visuomotor transformation between the controller and the screen and mapped kinematic information, computed in either coordinate system (controller or video), to the audio channel, in order to investigate which information was more relevant to the user.

**Results:**

Task-related audio feedback significantly improved performance with respect to visual feedback alone, whilst error-related feedback did not. Secondly, performance in audio tasks was significantly better with respect to the equivalent sensory-substituted visual tasks. Finally, with respect to visual feedback alone, video-task-related sound feedback decreased the tracking error during the learning of a novel visuomotor perturbation, whereas controller-task-related sound feedback did not. This result was particularly interesting, as the subjects relied more on auditory augmentation of the visualized target motion (which was altered with respect to arm motion by the visuomotor perturbation), rather than on sound feedback provided in the controller space, i.e., information directly related to the effective target motion of their arm.

**Conclusions:**

Our results indicate that auditory augmentation of visual feedback can be beneficial during the execution of upper limb movement exercises. In particular, we found that continuous task-related information provided through sound, in addition to visual feedback can improve not only performance but also the learning of a novel visuomotor perturbation. However, error-related information provided through sound did not improve performance and negatively affected learning in the presence of the visuomotor perturbation.

## Background

Over the past two decades there has been a rapid increase in the number of research groups and companies developing robotic devices for assisting movement rehabilitation of persons with disabilities (see reviews [1-6]). A variety of assistive control strategies have been designed (see review [7]), including: robots that move limbs rigidly along fixed paths, robots that assist only if the patient’s performance fails to stay within some spatial or temporal boundary, and soft robots that form a model of the patient’s weakness. Mechanical devices for rehabilitation are, in fact, designed to interact with the human guiding the upper limb through repetitive exercises based on a stereotyped pattern, and providing force feedback for sensorimotor type rehabilitative training [8].

Recent reviews on the first Randomized Controlled Trials (RCTs) of upper-limb robot-assisted rehabilitation outlined that clinical results are still far from being fully satisfactory [9-11]. Indeed, even though motor recovery is usually greater in robot-assisted groups than in control groups, only a few studies on acute and sub-acute phase robotic rehabilitation reported higher gains with respect to controls at the functional level (i.e., in the activities of daily living) [12]. These results suggest that the therapy devices, exercises and protocols developed so far still need to be improved and optimized [13].

Probably the most fundamental problem that robotic movement therapy must address in order to progress, is the lack of knowledge on how motor learning during neuro-rehabilitation works [14]. Many experimental results suggest that after local damage to the motor cortex, rehabilitative training with active engagement of the participant can shape subsequent reorganization in the adjacent intact cortex, and that the undamaged motor cortex may play an important role in motor recovery [15,16]. There is also evidence that kinematic error drives motor adaptation [17-19] and moreover, that humans adapt to robot-generated dynamic environments in a way that appears to minimize a cost function in terms of both error and effort [20].

It is also still not clear how the central nervous system combines different kinds of simultaneous feedback, such as proprioceptive and visual information or haptic feedback. It is known that visual and proprioceptive feedback may be combined in fundamentally different ways during trajectory control and final position regulation of reaching movements [21] and that when estimating the position of the arm, the brain selects different combinations of sensory inputs based on the computation in which the resulting estimate will be used [22]. Moreover, people tend to make straight and smooth hand movements when reaching for an object [23]. These trajectory features being resistant to perturbation, and proprioceptive, as well as visual feedback may guide the adaptive updating of motor commands enforcing this regularity. Morris et al. [24] found that recall following visuohaptic training, is significantly more accurate than recall following visual or haptic training alone, although haptic training alone is inferior to visual training alone. However, the precise ways that mental engagement, repetition, kinematic error, and sensory information in general, translate into a pattern of recovery, is not well defined for rehabilitation [14].

Audio is used in many rehabilitation systems with the purpose of motivating patients in their performance, either by reinforcing the realism of the virtual reality environment [25-27], or by providing information to guide the execution of the task [28,29]. However, the potential of auditory feedback in rehabilitation systems is largely underestimated in the current literature [30]. Maulucci et al. [31] used audio feedback to inform stroke subjects on the deviation of their hand from the ideal motion path and found that the auditory feedback training improved performance. There is also evidence that the effect of sound feedback in reaching tasks after chronic stroke, depends on the hemisphere which was damaged by the stroke [32], and that a proper sound may help individuals in learning a motor task [33,34], or in remaining engaged during robot assisted training in the presence of distractions [35].

The main goal of our research is to investigate the role of sound in motor learning and motor control, as additional sensory information to the visual and proprioceptive modalities, with the aim of incorporating optimized real-time auditory feedback related to one or more variables (i.e., position error or velocity) in augmented-feedback robotic rehabilitation systems. An incentive for this research is given by the observation that audio, just like video, is more direct and requires less attention than proprioception as an input modality [36]. Thus, auditory feedback may be potentially relevant, not only as a stimulation to augment a patient’s engagement and motivation, but also as additional or straightforward substitutive information, with respect to video, to improve performance and learning.

In this paper we present the results of three experiments on healthy subjects, in which the effects of different auditory feedback modalities during the execution of tracking motion exercises were investigated. In the first experiment, we studied whether continuous task-related auditory feedback can help to improve performance more than error-related audio feedback, or visual feedback alone. In the second experiment, we used sensory substitution [37] to compare different types of auditory feedback with equivalent visual feedback, in order to find out whether mapping the same information on a different sensory channel (the visual channel), yielded comparable effects with those gained in the first experiment. In the third experiment, we applied a continuously changing visuomotor transformation between the controller and the screen and mapped kinematic information, computed in either coordinate system (controller or video), to the audio channel, in order to investigate which information was more relevant to the user.

## Experiment #1

### Methods

#### Subjects

A total of 20 healthy subjects participated in the experiment. They were Caucasian, aged between 21 and 29 years old (mean age 22.95±1.99), 55% male and 45% female, and right-handed. All the participants had normal vision with no color blindness and self-reported no hearing problems. Written informed consent for publication of this report and any accompanying images was obtained from the subjects involved in all experiments. All experiments were performed with the ethical approval of the Scientific Commission of the University of Padua.

#### Experimental setup

**Figure 1:**
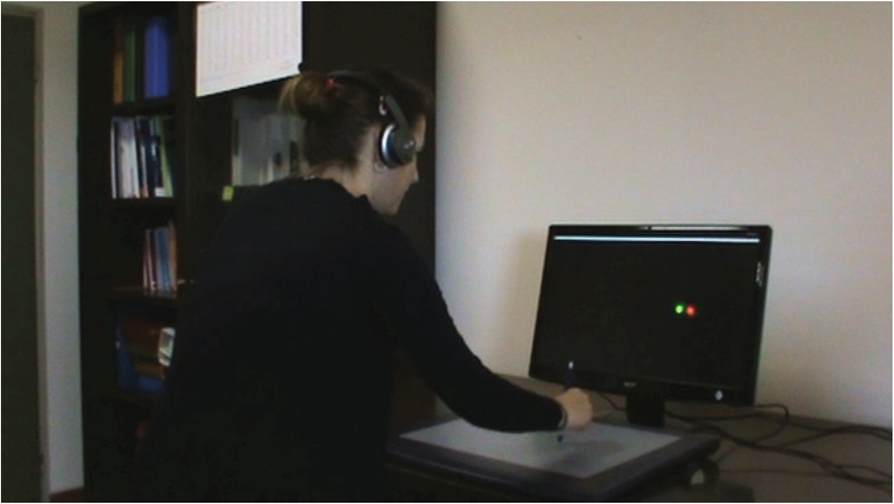
Healthy subject during a trial of Experiment #1.

**Figure 2:**
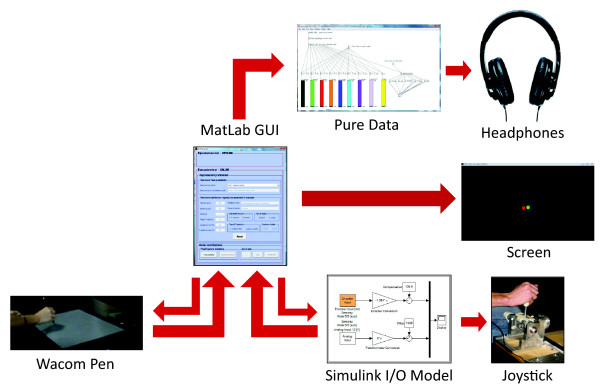
**Scheme of the experimental setup representing the main functional elements and data exchange between subsystems. ***The Wacom pen was used in Experiment #1; the joystick in Experiment #2 and Experiment #3.*

The experimental apparatus consisted of a Wacom pen as the input device (controller), a pair of common headphones that presented audio feedback to the user, and a full HD screen backed by a blank wall for video feedback (see Figure 1). A simple scheme of the system’s architecture is shown in Figure 2; the Simulink model and the joystick were not used in this experiment. The main application was implemented in MatLab.

Two color-filled, 25-pixel-radius dots were displayed on the screen; one representing the controller’s position (green dot) and the other, the target’s position (red dot). The target’s motion consisted of a left-right-left horizontal movement with a minimum-jerk velocity profile. Two different types of target motion were envisaged: 

• a *fixed-length* profile where the length of each left-right-left movement cycle was set to 60% of the screen size for all iterations within the same session, corresponding to a range of motion for the subject’s hand of nearly 300 *mm*;

• a *random-length* profile, where for each iteration the length of the segment pseudo-randomly varied from 20 to 90% of the screen size. At the end of the session, the total distance traveled by the target was the same as in the first case.

Audio feedback was developed in Pure Data, a real-time audio synthesis platform. The target’s and subject’s data (positions and velocities in the *X* and *Y * directions) were sent in real-time to Pure Data using the OSC (Open Sound Control) protocol. Two different types of audio feedback were designed: 

• *task-related* audio feedback, simulating the sound of a rolling ball;

• *error-related* audio feedback, performing formant synthesis of voice.

For the task-related feedback, the velocity of the target was applied as a simple gain factor onto the output of a pink noise generator filtered through a bandpass filter with a 200-Hz center frequency and Q factor equal to 7. For the error-related feedback, the position errors between the indicator and target in both axes were used to control the parameters of a formant synthesis patch. Specifically, the X-axis position error was mapped onto the amplitude and the fundamental frequency of a synthetic vocalized sound, whilst the Y-axis position error controlled the formants (i.e., the couple of frequencies that produce a vowel) of the sound. Both audio feedbacks were processed through a binaural spatialization filter, which renders the angular position of the sound source relative to the subject in the horizontal plane.

#### Experimental protocol

Each participant was asked to complete six different tasks. For each task, the subject had to draw a trajectory onto the tablet with the pen in order to follow the target on the screen. The six tasks were: 

• Task A: fixed-length trajectory without sound feedback

• Task B: random-length trajectory without sound feedback

• Task C: fixed-length trajectory with task-related sound feedback

• Task D: random-length trajectory with task-related sound feedback

• Task E: fixed-length trajectory with error-related sound feedback

• Task F: random-length trajectory with error-related sound feedback

Each task lasted 80 seconds and consisted of 13 repetitions of the left-right-left movement cycle. During each task, the target and subject’s position and velocity were sampled at a frequency of 300 *Hz*. After a first warm-up task showing no target, during which the subject could get acquainted with the tablet, she or he executed all tasks in a randomly-generated sequence. During the three seconds preceding the beginning of each task, a countdown was simulated through a sequence of three tonal beeps.

#### Data analysis

For each participant, the integral of the relative velocity (i.e., the difference between the subject’s and target’s velocities) and the weighted position error along the horizontal direction (X-axis) were measured. Each measure was calculated for every left-right and right-left segment, then it was averaged over the whole task.

The *integral of relative velocity* for the *k*−*th*segment is defined as:

(1) $$R_{v}(k)=\frac{1}{L_{k}}\overset{t_{k+1}}{\underset{t_{k}}{\int }}|{\vec{v}}_{r}|\mathrm{dt}$$

where: $\left|{\vec{v}}_{r}|=|{\vec{v}}_{s}-{\vec{v}}_{t}\right|$ is the norm of the relative velocity vector, *L*_*k*_is the length of segment *k*, whereas *t*_*k*_ and *t*_*k* + 1_ are the beginning and end times of the segment. *R*_*v*_was calculated using the rectangle method:

(2) $$\overset{N}{\underset{h=1}{\sum }}\frac{\sqrt{{\left(v_{x,s}(h)-v_{x,t}(h)\right)}^{2}+{\left(v_{y,s}(h)-v_{y,t}(h)\right)}^{2}}\cdot \mathrm{dt}}{L_{k}}$$

where N is the number of samples in the segment. The *R*_*v*_parameter measures the extra distance traveled by the subject while following the target, accounting for the movements made by the subject to correct tracking errors. A null value of this metric indicates that the velocity profile of the target has been exactly reproduced by the subject, even though the average position error (in terms of a constant offset measured by the second metric) may be not null.

The position error along the X-axis was weighted with the sign of target velocity and normalized to the target radius *R*. The *average weighted position error* for segment *k* is defined as:

(3) $$e_{x}(k)=\frac{1}{N}\overset{N}{\underset{h=1}{\sum }}\frac{\left(x_{s}(h)-x_{t}(h)\right)\cdot \mathrm{sign}\left(v_{x,t}(h)\right)}{R}.$$

This formula takes into account the direction of motion of the target, thus showing whether the subject leads (positive error) or lags (negative error) the target during the exercise. A null value in this metric indicates that the subject had an average null delay with respect to target motion, even though the distance traveled around the target (which is measured by the first metric) may be not null.

The D’Agostino and Pearson omnibus normality test revealed a Gaussian distribution for both the integral of relative velocity and the weighted position error. These performance measures were compared for initial differences in SPSS through a two-way within-subjects analysis of variance (ANOVA), with feedback and trajectory as within factors. When no interaction was found, pairwise post-hoc comparisons (Bonferroni’s test) were performed to evaluate the main effects more accurately. The significance level for the data analysis was set to *α*=0.05.

### Experimental results

The measures of the subjects’ performance indicated a lack of interaction between the two factors (i.e., feedback and trajectory), allowing a separate analysis of the effects due to these parameters (integral of relative velocity: *F*(2,38)=0.485, *p*=0.540, $\eta_{p}^{2}=0.025$; average weighted position error: *F*(2,38)=0.315, *p*=0.661, $\eta_{p}^{2}=0.016$).

**Figure 3:**
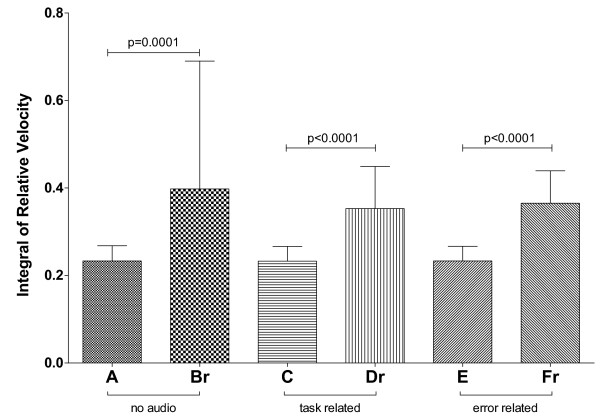
**Integral of relative velocity for the six tasks of Experiment #1.** Significant pairwise post-hoc comparisons (Bonferroni’s test) are indicated, after performing a two-way within-subjects ANOVA (feedback ∗ trajectory: **F(2,38)=0.485**, **p=0.540**, $\eta_{p}^{2}\;=0.025$; trajectory: **F(1,19)=44.095**, **p<0.0001**, $\eta_{p}^{2}\;=0.699$; feedback: **F(2,38)=0.173**, **p=0.734**, $\eta_{p}^{2}\;=0.009$).

The main result of the statistical analysis on the integral of relative velocity was that, as one may expect, the fixed-length task is always better executed than the corresponding random-length task (*F*(1,19)=44.095, *p*<0.0001, $\eta_{p}^{2}=0.699$), regardless of the audio modality employed (see Figure 3): the subjects made significantly greater corrections in the random-length tasks with respect to the corresponding fixed-length tasks for every modality.

On the other hand, no statistically significant difference was found, in terms of extra distance traveled around the target, when the audio modality was changed whilst keeping the same trajectory type (*F*(2,38)=0.173, *p*=0.734, $\eta_{p}^{2}=0.009$), indicating that the audio modality did not affect the number of corrections made by the subject whilst tracking the target.

**Figure 4:**
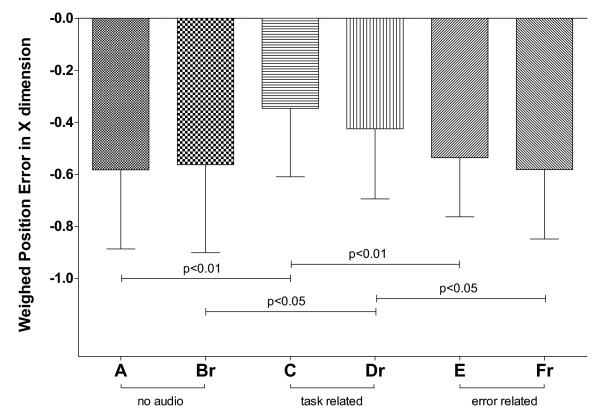
**Average weighted position-error for the six tasks of Experiment #1.** Significant pairwise post-hoc comparisons (Bonferroni’s test) are indicated, after performing a two-way within-subjects ANOVA (feedback ∗ trajectory: **F(2,38)=0.315**, **p=0.661**, $\eta_{p}^{2}\;=0.016$; trajectory: **F(1,19)=0.635**, **p=0.435**, $\eta_{p}^{2}\;=0.032$; feedback: **F(2,38)=11.207**, **p<0.0001**, $\eta_{p}^{2}\;=0.371$).

The statistical analysis on the average weighted position error revealed that, in terms of tracking delay, there is no significant difference between the fixed and random length tasks within the same audio feedback modality (Figure 4, *F*(1,19)=0.635, *p*=0.435, $\eta_{p}^{2}=0.032$), indicating that the trajectory type did not affect the average tracking delay.

However, in this case the feedback modality influenced performance (*F*(2,38)=11.207, *p*<0.0001, $\eta_{p}^{2}=0.371$). In fact, task C presented a significantly smaller negative error with respect to tasks A and E (both *p*<0.01), whilst task D did the same with respect to B and F (both *p*<0.05). In other words, task-related audio feedback (C and D) helped the subjects to significantly reduce average tracking delay when compared with error-related audio feedback (E and F) and to no audio (A and B), both in the easier (fixed length) and in the more complex (random length) tasks.

## Experiment #2

### Methods

#### Subjects

A total of 22 healthy subjects participated to the experiment (mean age 23±1.66, 81.8*%* male and 18.2*%*female). They were caucasian and right-handed, except for one subject who was left-handed. All the participants had normal vision with no color blindness, and self-reported no hearing problems.

#### Experimental setup

**Figure 5:**
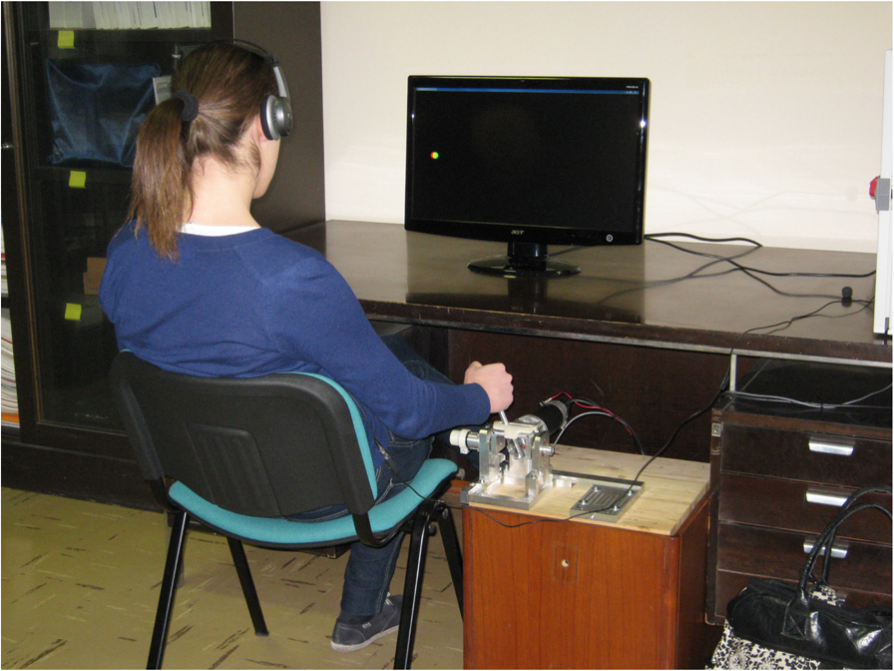
Healthy subject during a trial of Experiment #2.

The experimental setup, shown in Figure 5, was identical to that of Experiment #1 except for the input device; a 2-degrees-of-freedom passive joystick [38] monitored through a Sensoray data acquisition board and a Simulink model (see Figure 2 again).

As in the previous experiment, we represented on the screen two color-filled dots; one for the controller (green dot) and the other for the target (red dot), and we asked participants to perform a tracking exercise along the horizontal direction of the screen. In this experiment, all tasks shared a *fixed-length* trajectory with a minimum-jerk velocity profile, corresponding to a range of motion for the subject’s hand of 150 *mm*.

Three audio feedback modalities were implemented in Pure Data: 

• *task-related* audio feedback, simulating the sound of a rolling ball;

• *error-related* audio feedback, performing formant synthesis of voice;

• *velocity-error-related* audio feedback, simulating DJ scratching.

The first two modalities were identical to those of Experiment #1. Velocity-error-related audio feedback was designed as a cubic polynomial profile of the X-axis velocity error applied onto the output of a pink noise generator filtered through a bandpass filter, set up as in the task-related audio signal. In addition, a dead zone and a sign control were added to activate feedback only in the presence of medium-to-large errors, and when the controller was moving away from the target. This audio feedback was binaurally spatialized, as in the other two modalities.

Three alternate visual (color) feedback modalities, substituting each of the acoustic feedbacks, were also implemented. This was obtained by means of a progressive alteration of the screen’s background color, fading from black to light blue proportionally to the mapped quantity (i.e., X-axis target velocity, X-axis position error and X-axis velocity error).

#### Experimental protocol

Each participant was asked to complete seven different tasks. For each task, the participant had to move the joystick with the aim of following the target on the screen. The tasks were: 

• Task A: fixed-length trajectory with no audio and no color feedback

• Task B: fixed-length trajectory with position-error-related color feedback

• Task C: fixed-length trajectory with velocity-error-related color feedback

• Task D: fixed-length trajectory with task-related color feedback

• Task E: fixed-length trajectory with position-error-related audio feedback

• Task F: fixed-length trajectory with velocity-error-related audio feedback

• Task G: fixed-length trajectory with task-related audio feedback

Each task lasted about 90 seconds and consisted of 15 repetitions of the left-right-left movement cycle.

Each subject executed all the tasks in a randomly-generated sequence, after an initial warm-up task without the target, where the subject could get acquainted with the device. During the three seconds preceding the beginning of each task, a countdown was simulated through a sequence of three tonal beeps.

#### Data analysis

For this experiment we calculated the same metrics as in Experiment #1. Four participants who misinterpreted one or more color feedback tasks, were excluded from the analysis.

The D’Agostino and Pearson omnibus normality test revealed a Gaussian distribution for all tasks and metrics, whereas a Grubbs’ test recognized 2 outliers that were discarded. These metrics were compared for initial differences in SPSS through a one-way repeated measures analysis of variance (ANOVA). When ANOVA indicated significant differences, post-hoc tests (Bonferroni’s test) were performed to examine them in detail. The significance level for the data analysis was set to *α*=0.05.

### Experimental results

The measures of subjects’ performance differed depending on the feedback modality (integral of relative velocity: *F*(6,90)=7.558, *p*<0.0001, $\eta_{p}^{2}=0.335$; weighted position error: *F*(6,90)=24.07, *p*<0.0001, $\eta_{p}^{2}=0.616$).

**Figure 6:**
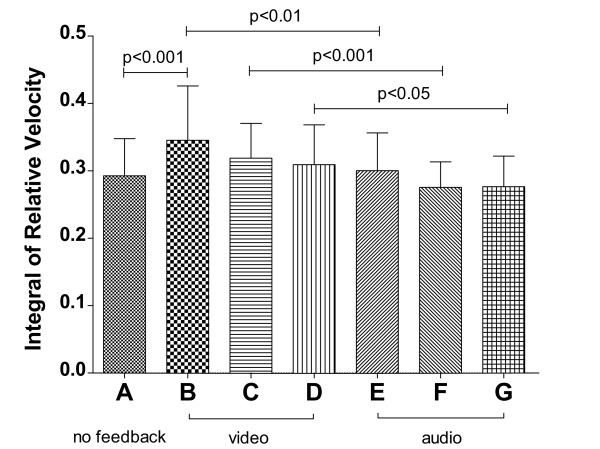
**Integral of relative velocity measure for the seven tasks of Experiment #2.** Significant pairwise post-hoc comparisons (Bonferroni’s test) are indicated, after performing a one-way repeated-measures ANOVA (**F(6,90)=7.558**, **p<0.0001**, $\eta_{p}^{2}\;=0.335$).

The comparison of the integral of the relative velocity between tasks A and B in Figure 6, shows that the addition of the error-related color feedback increases the extra total distance traveled by the subject (*p*<0.001). Moreover, each color feedback modality (B, C and D) induces significantly greater trajectory corrections with respect to the corresponding substituted audio modality (E, F and G; *p*<0.01, *p*<0.001 and *p*<0.05 respectively). Concerning audio tasks, the results confirmed those found in Experiment #1 (no significance on this metric if compared with the first task), including the new sound modality (velocity-error related audio): the audio feedbacks do not significantly alter the extra total distance traveled by the subject whilst tracking the target (*p*>0.05).

**Figure 7:**
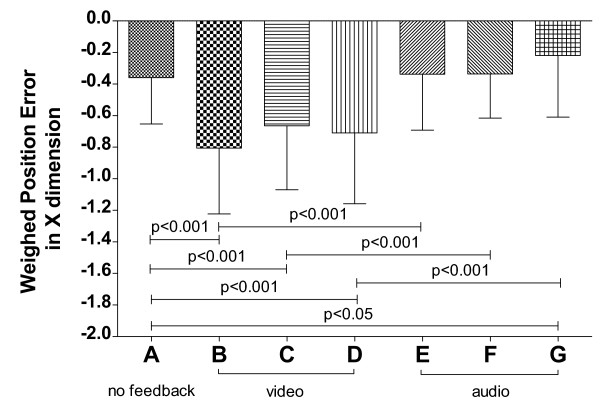
**Average weighted position-error for the seven tasks of Experiment #2.** Significant pairwise post-hoc comparisons (Bonferroni’s test) are indicated, after performing a one-way repeated-measures ANOVA (**F(6,90)=24.070**, **p<0.0001**, $\eta_{p}^{2}\;=0.616$).

Figure 7 shows the results of average weighted position error. All background-color alterations increase tracking delay if compared with task A (*p*<0.001 each), and with respect to the corresponding substituted audio modality (*p*<0.001 each). Concerning audio tasks, the results confirmed those found in Experiment #1 for task-related and position-error-related audio (*p*<0.05 and *p*>0.05), whereas velocity-error-related audio yields comparable results to task A in terms of average tracking delay (*p*>0.05), as position-error-related audio does.

One interesting point is that auditory feedback is effective independent of the controller used in the experiments: two very different input devices (pen tablet and joystick) yielded totally comparable results.

## Experiment #3

### Methods

#### Subjects

A total of 47 healthy subjects participated in the experiment (mean age 24.04±2.77, 78.7*%* male and 21.3*%*female). They were caucasian and right-handed, except for two subjects who were left-handed. All the participants had normal vision and self-reported no hearing problems.

#### Experimental setup

In this experiment we employed the same hardware and software equipment used in Experiment #2. The target movement displayed on the screen had a minimum-jerk velocity profile, in which the length of each segment: 

• in the first phase (*warm-up task*) was kept constant as in Experiment #2;

• in the second phase (*visuomotor transformation task*) pseudo-randomly varied from 20 to 90% of screen size; in addition, the scale between the video and the joystick changed at each iteration in such a way that the required motion of the joystick remained fixed along all segments (as in the warm-up task): owing to the alteration of the video-joystick scale introduced, the random-length motion of the target visualized in this phase corresponded to the same fixed-length target motion of the subject’s hand used in the warm-up.

**Figure 8:**
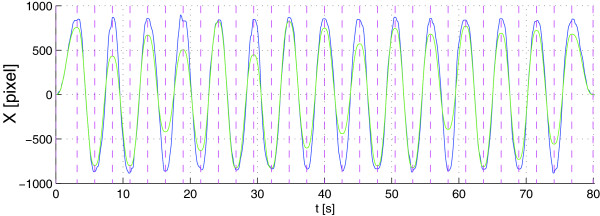
**X position versus time of target (green line) and subject (blue line) in one representative trial of the visuomotor transformation task of Experiment #3.** Subject position has been converted into pixels for the purpose of this chart. Dashed violet lines indicate the beginning of each trajectory segment. It can be seen that despite the variable amplitude of target motion, the subject tends to make a fixed amplitude motion due to the presence of the visuomotor transformation.

Figure 8 depicts the X position versus time of the target (green line) and of the subject (blue line) in one representative run of the visuomotor transformation task. It is clearly shown in the figure that, despite the variable amplitude of target motion, the subject tends to make a fixed amplitude motion, due to the presence of the visuomotor transformation. We can summarize by saying that, in this modality the target motion of the arm had a fixed length, whilst the motion of the target displayed on the screen had a randomly-variable length.

Three different audio feedbacks were used: 

• *error-related* audio feedback, performing formant synthesis of voice; in this modality, the mapped quantity was the position error on the X-axis, measured on the screen;

• *video-task-related* audio feedback, simulating the sound of a rolling ball by mapping the target velocity in screen scale;

• *joystick-task-related* audio feedback, simulating the sound of a rolling ball by mapping the target velocity in joystick scale.

By using the last two modalities, we intended to test whether the effectiveness of task-related audio in reducing the average tracking error, as observed in Experiment #1 and Experiment #2, was induced by an augmented description of the visualized task, or by audio rendering of the target motion of the arm.

#### Experimental protocol

Subjects were randomized into four groups, based on the kind of feedback provided during the experiment: 11 subjects received no sound feedback (NF), 12 subjects received error-related sound feedback (ER), 12 subjects received video-task-related sound feedback (TR-V), and 12 subjects received joystick-task-related sound feedback (TR-J). Subjects were asked to follow the target on the screen by moving the joystick. No information on the visuomotor transformation was provided to the subjects.

The warm-up task was made of 20 repetitions of the fixed-length, fixed scale target trajectory. After a 5 minute rest, a sequence of three tonal beeps signaled the beginning of the visuomotor transformation task. This task, which lasted about 80 seconds, consisted of 15 repetitions of the left-right-left movement cycle with random-length, visually altered trajectory.

#### Data analysis

We calculated the same metrics as in Experiment #1 and Experiment #2, using the visual scale, and performed a comparison between the groups in the visuomotor transformation task.

The D’Agostino and Pearson omnibus normality test revealed a Gaussian distribution for all groups and metrics. In addition, a total of 6 outliers (1 for NF and ER; 2 for TR-V and TR-J) were discarded after performing a Grubbs’ test. Thus, a one-way between-subjects analysis of variance (ANOVA) was performed in SPSS to compare each metric among the different groups (i.e., among different feedback modalities). When ANOVA indicated significant differences, pairwise post-hoc tests (Bonferroni’s test) were performed. The significance level for the data analysis was set to *α*=0.05.

### Experimental results

**Figure 9:**
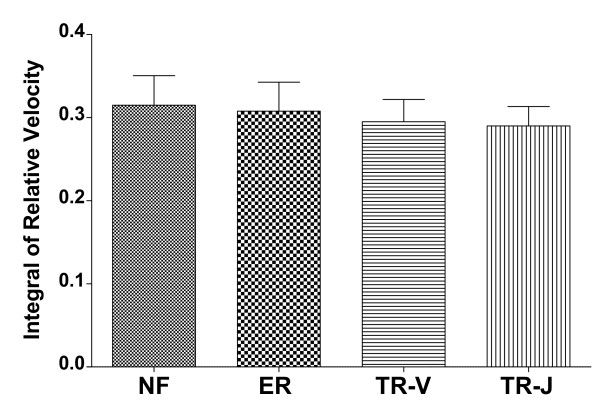
**Integral of relative velocity for Experiment #3.** By performing a one-way between-subjects ANOVA, the feedback modality results in a way not affecting the traveled extra distance (**F(3,37)=1.926**, **p=0.142**, $\eta_{p}^{2}\;=0.135$).

The histogram of the integral of relative velocity, shown in Figure 9, reports no statistically significant difference between groups (*F*(3,37)=1.926, *p*=0.142, $\eta_{p}^{2}=0.135$), i.e., the extra total distance traveled by the subject’s hand during the task is not influenced by the audio feedback provided, and is comparable with that of group NF (no audio feedback).

**Figure 10:**
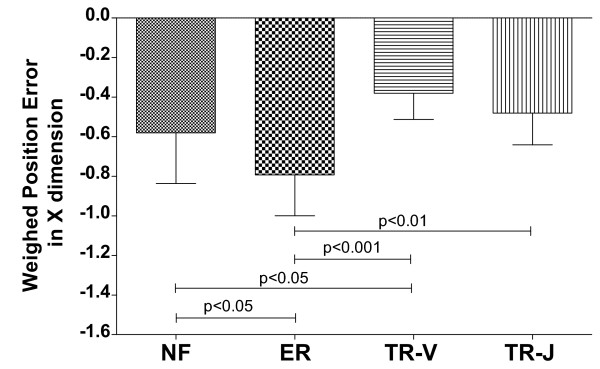
**Average weighted position-error for Experiment #3.** Significant pairwise post-hoc comparisons (Bonferroni’s test) are indicated, after performing a one-way between-subjects ANOVA (**F(3,37)=10.100**, **p<0.0001**, $\eta_{p}^{2}\;=0.450$).

Regarding the average weighted position error (Figure 10), the tracking delay was influenced by the feedback modality (*F*(3,37)=10.100, *p*<0.0001, $\eta_{p}^{2}=0.450$). In particular, we can observe that in presence of the visuomotor transformation, the error-related audio feedback yields significantly greater average tracking delays with respect to all other modalities (*p*<0.05, *p*<0.001, *p*<0.01, respectively compared with NF, TR-V and TR-J). In other words, providing position-error related information through sound, despite being substantially equivalent to the absence of audio feedback in #1 and #2, may be detrimental during the learning of a novel visuomotor transformation.

On the other hand, providing task-related information through sound during learning of a novel visuomotor transformation can be beneficial, if the auditory information is consistent with that provided by visual feedback, yielding reduced average tracking delay with respect to having no audio feedback (*p*<0.05). In contrast, providing auditory information related to the expected arm movement is not likely to bring benefits in the presence of a novel visuomotor transformation (*p*>0.05).

The statistics of all experiments are summarized in Table 1.

**Table 1:** Statistical analysis on both the integral of relative velocity (*R*_*v*_) and the weighted position error (*e*_*x*_) for the three experiments

**Exp.**	**Metric**	**Test**	**Comparisons**	**Statistical results**		
Exp. 1	*R*_*v*_	ANOVA	Fdbk ∗ Traj	*F*(2,38)=0.485	*p*=0.540	$\eta_{p}^{2}=0.025$
			Traj	*F*(1,19)=44.095	*p*<0.0001	$\eta_{p}^{2}=0.699$
			Fdbk	*F*(2,38)=0.173	*p*=0.734	$\eta_{p}^{2}=0.009$
	*e*_*x*_	ANOVA	Fdbk ∗ Traj	*F*(2,38)=0.315	*p*=0.661	$\eta_{p}^{2}=0.016$
			Traj	*F*(1,19)=0.635	*p*=0.435	$\eta_{p}^{2}=0.032$
			Fdbk	*F*(2,38)=11.207	*p*<0.0001	$\eta_{p}^{2}=0.371$
		Bonferroni	NF-TR		*p*=0.001	
			NF-ER		*p*=1.000	
			TR-ER		*p*=0.001	
Exp. 2	*R*_*v*_	ANOVA	Fdbk	*F*(6,90)=7.558	*p*<0.0001	$\eta_{p}^{2}=0.335$
		Bonferroni	A-B		*p*<0.001	
			B-E		*p*<0.01	
			C-F		*p*<0.001	
			D-G		*p*<0.05	
			A-F		*p*>0.05	
	*e*_*x*_	ANOVA	Fdbk	*F*(6,90)=24.070	*p*<0.0001	$\eta_{p}^{2}=0.616$
		Bonferroni	A-B		*p*<0.001	
			A-C		*p*<0.001	
			A-D		*p*<0.001	
			B-E		*p*<0.001	
			C-F		*p*<0.001	
			D-G		*p*<0.001	
			A-F		*p*>0.05	
Exp. 3	*R*_*v*_	ANOVA	Fdbk	*F*(3,37)=1.926	*p*=0.142	$\eta_{p}^{2}=0.135$
	*e*_*x*_	ANOVA	Fdbk	*F*(3,37)=10.100	*p*<0.0001	$\eta_{p}^{2}=0.450$
		Bonferroni	ER-NF		*p*<0.05	
			ER-TR-V		*p*<0.001	
			ER-TR-J		*p*<0.01	
			NF-TR-V		*p*<0.05	
			NF-TR-j		*p*>0.05	

The factors investigated in these experiments are either the feedback modality (Fdbk) or the trajectory profile (Traj).

## Discussion and conclusion

The results of our experiments confirm that auditory augmentation of visual feedback can be beneficial to the user’s performance in upper limb movement tasks, even in the presence of a novel visuomotor transformation. In particular, the addition of a secondary sensory channel that faithfully represents the information provided by the visual channel helps the user in having a stronger perception of the task, allowing for improved sensory-motor coordination. This result lies in accordance with [39], which states that coordination variability with more than one sensory modality is smaller than with one modality only; suggesting that the performer can easily manage the integration of visual and auditory information online during task execution, thus tending to optimize the signal statistics. However, providing the same information on task or error through vision does not bring about an upgrade in performance (Experiment #2), suggesting that visual information cannot be augmented through the same channel in the experienced motion tracking tasks. In fact, replacing auditory feedback with a background color transformation on the screen led to results that are even worse than having the original visual feedback alone. This finding indicates that, in our tests, the visual channel was already saturated by the target following task, so that the background color variation turned out to be a distraction, rather than useful additional information for the user. Instead, two separated information channels (visual and auditory in our experiments), if properly coordinated, work in a parallel fashion and can contribute to performance enhancement, even in the presence of visuomotor perturbations.

The rolling ball paradigm for the task-related audio used in the experiments, is obviously included in this class of continuous auditory cues, being a straightforward and intuitive means of providing velocity profiles through the auditory channel. Task-related auditory feedback proved to be effective in reducing the average tracking error, even though it did not affect the number of trajectory corrections made by the subject whilst attempting to follow the target (integral of relative velocity). Such a result is consistent with the observation that this audio modality can be considered as a feed-forward input for the subject’s motor control. Conversely, providing error-related information through sound in the presence of visual feedback (through both formant synthesis reflecting position error and scratching effects reflecting velocity error, as in Experiment #2) did not affect tracking performance. This result may be explained by considering that error-related audio presents redundant information with respect to the visual modality, rather than providing an augmentation of the visual information available to the user. In addition, one may argue that the subject may expect to receive, or elaborate error related information from video rather than from the auditory sensory channel, and this may lead the subject to disregard the information received through sound.

Task-related audio was also effective in the presence of a visuomotor transformation explicitly designed to confuse the user (Experiment #3). In this context, the audio related to the visual scale, that was consistent with what the user actually saw on the screen, was more effective in reducing tracking delay with respect to the joystick-scale audio, which provided information on the effective target motion of the arm. This result is particularly interesting, as more correct information on desired arm motion was provided in the joystick-related modality, which in turn yielded worse results. In contrast, performance was improved by enhancing task information that was inconsistent with the desired arm motion. This finding suggests that the subject tends to expect information on task, rather than on motor command from extrinsic feedback. Secondly, video-related audio provides additional information in accordance with the sensory channel onto which the user’s attention is already focused, following a visual dominance principle. In other words, the user manages to compensate for the mismatch between the two movement ranges by relying mostly on the visual feedback, yet the sensory augmentation given by the visual-scale auditory feedback contributes to increased performance with respect to the condition where the auditory channel is not used. Conversely, creating a conflict between the audio and video modalities leads the user to maintain attention focused onto the visual input [40], obtaining results comparable with those gained in absence of the audio signal. This is in agreement with [39], where it is stated that misleading or noisy feedback increases coordination variability, although saturating toward the level without feedback at most.

The results of the experiments presented in this paper indicate that the introduction of a straightforward and meaningful auditory signal can remarkably enhance performance during the execution of motion-tracking exercises. This is most likely due to the fact that provision of additional information through the auditory system allows parallel processing. Indeed, rather than acting as a confounding influence, sound feedback enhances visuo-motor control because it provides similar information [41]. We studied such an influence on healthy subjects first in order to investigate the normative response of the human motor system to auditory feedback.

The improvement of performance obtained from video-task-related audio in the presence of a continuously varying visuomotor perturbation, indicates that sound can be effective in supporting continuous learning of novel visuomotor transformations. Consequently, we hypothesize that a properly designed task-related audio signal, provided continuously to the user, may lead to enhanced learning in robot-assisted rehabilitation. The effects of feedback on motor performance and learning have been extensively discussed in the literature [6,42]. According to Timmermans et al. [6], rehabilitation technology should provide both knowledge of results, as well as knowledge of performance to the patient. In addition to this, we believe that the effects of providing task-related information through sound should be investigated more thoroughly. To date, although there have been attempts to use sound in a more sophisticated way, auditory feedback is underutilized in most robotic therapy systems, playing a role as background music or signifying only the task completion in most cases [30]. Nonetheless, understanding the real potential of audio in the rehabilitation context requires further investigation that will be addressed in future research.

## Competing interests

The authors declare that they have no competing interests.

## Authors’ contributions

All authors contributed equally to the design of experiments. Spagnol and Avanzini implemented the sound synthesis software. Rosati and Oscari leaded the experimental testing phase and performed data processing and statistical analysis. All authors read and approved the final manuscript.
